# Prevalence of Post-Traumatic Stress Disorder after Childbirth: A Tunisian Sample

**DOI:** 10.1192/j.eurpsy.2023.2423

**Published:** 2023-07-19

**Authors:** R. Ben Soussia, N. Faouel, W. Bouali, I. BlHadj, H. Bouchahda, L. Zarrouk

**Affiliations:** 1 Psychiatry Department, Tahar Sfar Hospital -Mahdia Tunisia; 2 Department of Psychiatry; 3 Psychiatry Department; 4 Tahar Sfar Mahdia Hospital; 5 Gynecology Department, Tahar Sfar hospital Mahdia, Mahdia, Tunisia

## Abstract

**Introduction:**

Childbirth is a period of transition for women, which can have several physiological and psychological repercussions on their lives.

**Objectives:**

To estimate the prevalence of postpartum post-traumatic stress disorder in a sample group of Tunisian women.

**Methods:**

This is a longitudinal prospective descriptive study carried out among women who were hospitalized for childbirth in the obstetrics gynecology department and those who consulted the outpatient perinatal consultation of the Tahar Sfar Mahdia hospital during a period of 7 months from March 15, 2020 to September 15, 2020. Data collection was based on a pre-established questionnaire determining the different socio-demographic and clinical characteristics.

The women’s psychometric assessment was conducted using a PCLS psychiatric scale for screening for post-traumatic stress disorder.

**Results:**

We collected 120 women with an average age of 28.3 ±5.3 years. Among our sample, two women had a history of depression (1.6%) and three participants had a history of an anxiety disorder (2.5%).Thirty-five patients (29.1%) had a pathological obstetric history. However, fifteen patients (12.5%) were hospitalized during their pregnancies. Eighty-seven patients (72.5%) expressed their anticipated fear of childbirth and one hundred two patients (85%) had a good marital relationship with good social support. 48.3% of deliveries were vaginal and 27.5% were by emergency cesarean section. Level three pain was expressed in 73.3% of deliveries. Psychometric assessment revealed a prevalence of PTSD at 5.8% with PTSD symptomatology in 18.4% of women.

**Conclusions:**

Postpartum post-traumatic stress disorder is a major public health problem that affects the healthy development of the newborn and the mental and physical recovery.

**Disclosure of Interest:**

None Declared

